# Prediction of Early Hospital Admission (≤24 Hours) After Stroke Using Machine Learning and Deep Learning: Multicenter Study From China

**DOI:** 10.2196/90852

**Published:** 2026-06-24

**Authors:** Qingjia Zeng, Jiachen Cui, Xinyu Fan, Dawei Li, Xiao Yang, Menghan Song, Shuangyang Niu, Yuhuan Wang, Yufeng Wang, Fubiao Huang, Yonghui Wang, Qiang Wu, Hongpu Hu

**Affiliations:** 1 Institute of Medical Information/Medical Library Chinese Academy of Medical Sciences & Peking Union Medical College Beijing, Beijing China; 2 Wolfson Centre for Prevention of Stroke and Dementia Department of Clinical Neurosciences University of Oxford Oxford, England United Kingdom; 3 School of Information Science and Engineering Shandong University Qingdao, Shandong China; 4 Department of Medical Statistics, School of Public Health Sun Yat-sen University Guangzhou, Guangdong China; 5 Shengli Oilfield Central Hospital Dongying, Shandong China; 6 Rehabilitation Center Qilu Hospital of Shandong University Jinan, Shandong China; 7 Qilu Hospital of Shandong University Dezhou Hospital Dezhou China; 8 People’s Hospital of Rehabilitation Weifang City Weifang, Shandong China; 9 Department of Occupational Therapy China Rehabilitation Research Center Beijing, Beijing China; 10 Faculty of Rehabilitation Capital Medical University Beijing, Beijing China; 11 Institute of Brain and Brain-Inspired Science Shandong University Jinan, Shandong China

**Keywords:** stroke, early hospital admission, multilayer perceptron, temporal validation, prehospital delay, multicenter study

## Abstract

**Background:**

Timely hospital admission is a prerequisite for effective acute stroke management, yet a substantial proportion of patients fail to reach medical facilities within the optimal therapeutic window. Existing prediction models often lack temporal robustness and clinical interpretability, limiting their utility in real-world, evolving health care systems.

**Objective:**

This study aimed to develop and temporally validate machine learning and deep learning models using multicenter clinical data to predict early hospital admission (≤24 h) after acute stroke.

**Methods:**

In this multicenter retrospective study, we analyzed routinely collected electronic medical record data from 1327 patients across 6 hospitals in China. We developed and compared 6 predictive models: logistic regression, support vector machine, random forest, multilayer perceptron (MLP), convolutional neural network, and long short-term memory, for early admission (≤24 h from symptom onset). Model training was performed on a train set (2019-2022), followed by independent temporal validation on a testing set (2023-2025). Model prediction performance was evaluated using discrimination metrics, sensitivity, and robustness under temporal distribution shift. Model interpretability was assessed using Shapley additive explanations.

**Results:**

A total of 1327 patients were included, of whom 821 were assigned to the train set and 506 to the independent temporal testing set. Among the 6 candidate models, the MLP showed the best overall performance in the independent temporal testing set, achieving an area under the receiver operating characteristic curve of 0.9020 (95% CI 0.8718-0.9283), sensitivity of 91.5%, specificity of 75.6%, and *F*_1_-score of 0.9033. Formal statistical comparisons showed that the MLP achieved significantly higher area under the receiver operating characteristic curve values than logistic regression, support vector machine, random forest, and one-dimensional convolutional neural network after false discovery rate correction, with a smaller but still statistically significant improvement over the long short-term memory. Calibration analysis further showed that the MLP had the most favorable overall calibration profile among the candidate models.

**Conclusions:**

In this multicenter Chinese cohort, the MLP showed favorable temporal performance for predicting early hospital admission after stroke. The model may support future risk stratification and targeted public health interventions, although further external validation and calibration refinement are needed before deployment-oriented use.

## Introduction

Stroke remains a catastrophic global health emergency and a leading cause of mortality and long-term disability. In 2021, the global burden of stroke reached approximately 11.9 million incident cases and 7.3 million deaths, with China accounting for roughly one-third of this burden [1,2]. The management of acute stroke is highly time-dependent, as reflected in the well-established concept that “time is brain.” Timely hospital presentation is therefore essential for effective stroke care, particularly because the benefit of reperfusion therapy is greatest when treatment is initiated as early as possible, especially within the first 3 hours after symptom onset [3-5]. However, in this study, we used a 24-hour threshold as a pragmatic classification boundary to distinguish relatively early versus delayed hospital admission in this retrospective multicenter dataset, rather than to define the optimal therapeutic window. Delayed presentation may place patients beyond the period of greatest treatment benefit and is associated with worse neurological outcomes, higher mortality, and greater health care burden [6]. Therefore, identifying the determinants of earlier hospital admission remains important for improving emergency response systems and patient prognosis.

Despite the implementation of stroke code systems and improvements in emergency medical services (EMS), a substantial proportion of patients with stroke still do not reach the hospital within 24 hours of symptom onset. Traditional epidemiological studies have identified multiple barriers to early hospital admission, including older age, rural residence, lower socioeconomic status, and limited awareness of stroke symptoms [7-9]. In addition, organizational and health-system factors, such as EMS activation, prenotification routines, ambulance use, and regional stroke-care strategies, may also influence timely access to hospital-based stroke care [10]. However, these studies have primarily approached delay from a social and behavioral perspective, with less attention to the complex clinical and biological profiles of patients at presentation. Emerging evidence suggests that factors such as functional impairment, cognitive status, and comorbidity burden may themselves shape help-seeking behavior and transport urgency [11,12]. Yet conventional statistical approaches often have limited ability to integrate these high-dimensional and potentially interacting variables into a unified predictive framework. This gap raises an important question: can routinely collected electronic medical record data be leveraged not only for retrospective description, but also for prediction of early versus delayed hospital admission after stroke using data-driven modeling approaches?

To decode the complex interactions between patient demographics and physiological parameters, medical research is increasingly turning to artificial intelligence [13-15]. Machine learning (ML) algorithms, such as random forest (RF), have demonstrated superior performance over conventional regression methods in triage decision-making by handling nonlinear data more effectively [16,17]. However, as clinical data becomes more complex, traditional ML may reach a performance plateau. Deep learning (DL), particularly the multilayer Perceptron (MLP), offers a further leap in analytical capability by capturing intricate, high-level abstractions that traditional algorithms miss [18-20]. Although DL has shown strong performance in several medical prediction tasks, its use for classifying early versus delayed hospital admission after stroke remains less well studied. In addition, the interpretability of DL models remains a practical concern in clinical settings, which motivated our use of Shapley additive explanations (SHAP) to examine how individual predictors contributed to model output.

Given the importance of timely hospital presentation after stroke, this study aimed to develop and temporally validate predictive models for early hospital admission using multicenter clinical data from 6 hospitals in China. We systematically compared 6 algorithms, including conventional ML models (logistic regression [LR], support vector machine [SVM], and RF) and DL models (MLP, convolutional neural network [CNN], and long short-term memory [LSTM] network). We hypothesized that DL models, particularly the MLP, would achieve stronger discrimination than conventional ML baselines under temporal validation in the independent temporal testing set. To enhance the clinical relevance of model evaluation, we adopted a chronological validation strategy in which models were developed using data from 2019-2022 and then evaluated in an independent temporal testing set from 2023-2025. By identifying the most suitable modeling approach, this study sought to provide an interpretable, data-driven tool for identifying patients at risk of delayed hospital presentation after stroke.

## Methods

### Study Population and Design

This multicenter retrospective study was conducted using data from 1327 patients with stroke across 6 hospitals in China, spanning from January 2019 to March 2025. The inclusion criteria were: (1) clinically confirmed ischemic stroke or intracerebral hemorrhage based on computed tomography or magnetic resonance imaging; (2) age ≥18 years; (3) admission to neurology or rehabilitation departments with stable medical status; and (4) documented time interval from symptom onset to hospital admission. Patients with subarachnoid hemorrhage were excluded because, although it is a hemorrhagic cerebrovascular event, it differs substantially from intracerebral hemorrhage in anatomical location, clinical presentation, pathophysiology, and acute management pathway. Patients were also excluded if stroke was not the primary reason for admission. The study protocol was approved by the Ethics Committee of the China Rehabilitation Research Center (approval number 2024-025-2). All records were de-identified before analysis, with direct personal identifiers removed prior to data integration and model development. Data transfer and use across participating centers were conducted in accordance with institutional approvals and applicable multicenter data-governance requirements.

To evaluate the temporal stability and model performance of the models against potential data drift, patients were partitioned chronologically:

1. Train set (n=821): Patients admitted between January 2019 and December 2022, used for feature selection, hyperparameter setting, and internal stratified 5-fold cross-validation. The train set was used exclusively for model development, and all candidate models were evaluated using the same cross-validation splits to ensure comparability across algorithms.
2. Independent temporal testing set (n=506): patients admitted between January 2023 and March 2025, reserved exclusively for final independent temporal validation and not used for feature selection, preprocessing parameter estimation, or model tuning.

Because this was a retrospective multicenter study based on consecutively collected real-world clinical records, no formal a priori sample size calculation was performed at the study design stage. In the train set, 821 patients were available for model development, and the final analytical pipeline retained 57 predictors. Based on the smaller outcome class in the train set (n=352), the corresponding events-per-variable ratio was approximately 6.18. We therefore interpret the present sample size as moderate rather than large for predictive modeling, particularly for flexible DL architectures, and we relied on independent temporal validation to provide an additional assessment of temporal performance under distribution shift.

### Outcome Measures and Candidate Predictors

The primary outcome was early hospital admission, defined as presentation to the hospital within 24 hours of symptom onset. Symptom-onset time was extracted from the retrospective electronic medical record as the first symptom-onset time documented at admission. In routine clinical practice, this time was generally recorded based on patient or family report. Admission time was defined as the first documented hospital arrival time in the electronic medical record. The 24-hour threshold was selected as a pragmatic classification boundary to distinguish relatively early versus delayed hospital admission within this retrospective multicenter dataset. The interval between symptom onset and hospital arrival was then used to classify early hospital admission (≤24 h) versus delayed admission (>24 h).

A total of 120 candidate predictors were extracted from the electronic medical record system and organized into five clinical domains: (1) demographics, including age, sex, residence (urban or rural), marital status, number of children, stroke subtype, occupation, and lifestyle factors such as smoking and alcohol use; (2) medical history, including preexisting diseases and family history of stroke or vascular disease in first-degree relatives; (3) admission comorbidities, classified according to *ICD-11* (*International Classification of Diseases, 11th Revision*) and International Classification of Functioning, Disability and Health (ICF) frameworks, including cardiovascular and metabolic disorders (eg, hypertension and diabetes), neurological impairments (eg, dysphagia and cognitive impairment), and acute complications (eg, pulmonary infection); (4) admission functional scores, including the Holden Functional Ambulation Classification and modified Barthel index; and (5) laboratory data, including more than 30 continuous markers covering glycemic status, electrolytes, renal and liver function, inflammatory markers, and cardiac enzymes. Laboratory variables were standardized before model input to improve comparability across predictors.

### Data Preprocessing and Feature Selection

An initial set of 120 candidate variables was extracted from the electronic medical record system. Variables with a missing rate >70% were excluded before model development; in total, 39 variables were removed on this basis. Among the retained variables, the overall proportion of missing data was 12.7%, and 43.5% of retained variables contained at least some missing values. For the primary analysis, missing values were imputed using *k*-nearest neighbors (KNN) imputation with k=5. The remaining variables were then preprocessed through encoding of categorical predictors and *z*-score standardization of continuous variables before model input. To avoid data leakage, within each fold of the stratified 5-fold cross-validation in the train set, missing-value imputation, normalization, and feature selection were fitted only within the fold-specific training subset and then applied to the corresponding validation subset.

To address high dimensionality and multicollinearity, feature selection was performed using least absolute shrinkage and selection operator (LASSO) regression with an L1 penalty. The optimal penalty parameter (λ) was determined by stratified 5-fold cross-validation within the development framework [21]. The final model retained 57 predictors with non-zero coefficients for subsequent model construction. To examine the robustness of feature selection, we additionally assessed selection stability across the cross-validation folds. The number of selected features showed moderate consistency across folds (mean 47.2, SD 9.4), and the mean pairwise Jaccard similarity of the fold-specific selected feature sets was 0.581 (SD 0.092), indicating moderate stability rather than arbitrary split-dependent selection. Several clinically relevant predictors, including cognitive impairment, diabetes, hypertension, and intracranial aneurysm, were selected consistently across folds.

After internal cross-validation, the final preprocessing pipeline, LASSO feature-selection procedure, and predictive models were refitted using the entire train set only. The fitted pipeline was then applied once to the independent temporal testing set for final temporal validation.

### Model Development and Internal Validation

To compare the predictive performance of conventional ML and DL approaches, we developed six candidate models: LR, SVM, RF, MLP, a 1D convolutional neural network (1D-CNN), and an LSTM network. All models were developed in the train set and evaluated using the same stratified 5-fold cross-validation splits. For the DL models, learning rate, batch size, and number of epochs were selected through pilot experiments within the train set, whereas conventional ML models were primarily implemented using prespecified or standard settings to provide strong and interpretable baselines. No formal early stopping procedure was used; instead, a fixed training schedule of 25 epochs was adopted for comparability across DL architectures.

LR: to establish a linear baseline, we implemented LR with L2 regularization using the liblinear solver. The model was fitted with class-weight adjustment to reduce the influence of class imbalance and a fixed random seed (random_state=42). Unless otherwise specified, the regularization strength was kept at the default setting (C=1.0), and the maximum number of iterations was 100 [22,23].

SVM: we implemented an SVM with a linear kernel to maintain a balance between model complexity and interpretability. Probability estimation was enabled through Platt scaling to support ROC-based evaluation. Class-weight adjustment and a fixed random seed (random_state=42) were applied, while the regularization parameter was kept at the default setting (C=1.0) [24-26].

RF: as an ensemble learning method based on bootstrap aggregation, RF was configured with 100 decision trees (n_estimators=100) and the Gini impurity criterion for node splitting. Other parameters were left at their library defaults, including max_depth=None, min_samples_split=2, and min_samples_leaf=1, with a fixed random seed (random_state=42) [27].

MLP: we designed a fully connected feed-forward neural network to capture nonlinear interactions among predictors. The final architecture consisted of an input layer followed by 2 hidden layers with 256 and 128 units, respectively, batch normalization, rectified linear unit activation, dropout, and a linear output layer. The model was trained using the Adam optimizer with a learning rate of 1×10^-3^, batch size of 32, and 25 epochs. These settings were selected through pilot experiments within the train set based on convergence behavior, training stability, and predictive performance [28,29].

1D-CNN: we adapted a 1D-CNN for tabular input by treating the predictor vector as a one-dimensional sequence. The final architecture comprised 2 convolutional layers (Conv1d(1,32), kernel size=3, padding=1; and Conv1d(32,64), kernel size=3, padding=1), followed by adaptive global average pooling and dropout. The network was trained using the Adam optimizer with a learning rate of 1×10^-3^, batch size of 32, and 25 epochs [30,31].

LSTM network: we included an LSTM model as an exploratory deep-learning comparator adapted to reshaped tabular input rather than as a genuine longitudinal sequence model. Input features were reshaped into a single-step format only to enable empirical comparison with an LSTM-based neural architecture; this design was not intended to model true temporal sequences. The architecture used a hidden dimension of 64 and 2 recurrent layers, followed by a fully connected layer with 128 units and dropout before the output layer. The model was trained using the Adam optimizer with a learning rate of 1×10^-3^, batch size of 32, and 25 epochs [32].

### Sensitivity Analyses

To assess the robustness of the preprocessing strategy, we conducted supplementary sensitivity analyses using alternative imputation methods, including KNN with *k*=3 and *k*=7, multiple imputation by chained equations (MICE), and mean imputation. Because the MLP showed the best overall predictive performance in the primary analysis, these additional imputation analyses were conducted using the MLP as the reference model. The results supported the use of KNN with *k*=5 as the primary strategy, as it provided the most favorable overall balance of discrimination and classification performance in the independent temporal testing cohort (Table S1 in Multimedia Appendix 1).

### Model Evaluation and Validation

Model performance was comprehensively assessed using discrimination, calibration, and decision-analytic measures. Discrimination was evaluated by accuracy, precision, sensitivity (recall), specificity, *F*_1_-score, and the area under the receiver operating characteristic curve (AUC-ROC). Calibration was assessed in the independent temporal testing set using the Brier score, calibration intercept, calibration slope, and calibration curves. To evaluate potential clinical utility beyond discrimination alone, decision curve analysis was performed by comparing each model with the treat-all and treat-none strategies across a range of threshold probabilities. In the primary analysis, binary classification metrics were calculated using the default probability threshold of 0.5 for all models. We additionally performed a threshold sensitivity analysis for the MLP model at representative probability cutoffs of 0.3, 0.4, 0.5, and 0.6.

To formally compare model performance, we conducted additional statistical analyses in the independent temporal testing set. The AUC of the MLP model was compared with that of each alternative model using the DeLong test. Paired classification errors at the primary operating threshold of 0.5 were compared using the McNemar test. For key performance metrics, including AUC, sensitivity, specificity, and *F*_1_-score, 95% CIs were calculated. To account for multiple pairwise comparisons, *P* values were adjusted using the false discovery rate (FDR) procedure.

To enhance the interpretability of the optimal model, SHAP analysis was performed for the positive class, defined as early hospital admission (≤24 h). We used GradientExplainer for the final MLP model, with the first 200 samples from the train set serving as the background dataset. SHAP values were computed on the final preprocessed model inputs; accordingly, feature values displayed in the SHAP beeswarm and dependence plots reflect the values used by the model after preprocessing rather than the raw variables recorded in the electronic medical records. For binary indicators such as dysphagia, the plotted values therefore represent the encoded model inputs used in the SHAP analysis, whereas continuous variables are shown on their standardized scale after *z*-score normalization. Global interpretation was summarized using SHAP beeswarm plots, and additional feature-specific dependence plots and representative local waterfall plots were used to examine the directional contribution of selected predictors at both the cohort and individual levels. To assess the robustness of the SHAP findings, we further repeated the analysis using alternative background samples, and the corresponding supplementary results are provided in Multimedia Appendix 1. All experiments were conducted with a fixed random seed of 42. The analyses were performed in Python 3.10 using PyTorch 1.13.1, scikit-learn 1.5.2, and SHAP 0.46.0. Model training was conducted on an NVIDIA GeForce RTX 4060 GPU. R software (R Foundation for Statistical Computing) was used for selected supplementary statistical analyses and figure preparation.

### Ethical Considerations

This study was performed in accordance with the principles of the Declaration of Helsinki. The study protocol received ethical approval from the Ethics Committee of the China Rehabilitation Research Center (approval number 2024-025-2). Given the retrospective design of the study and the use of de-identified medical records, the requirement for informed consent was waived by the ethics committee. All records were anonymized before analysis, and data handling and transfer were conducted in accordance with institutional approvals and applicable multicenter data-governance requirements. No financial or material compensation was provided to participants for their involvement in this study. Participation was entirely voluntary, and all procedures were conducted following relevant ethical guidelines.

## Results

### Baseline Characteristics

Of the included patients, 52.4% (n=696) arrived at the hospital within 24 hours of symptom onset, whereas 47.6% (n=631) presented later than 24 hours. The comprehensive baseline characteristics, stratified by admission timing, are detailed in Table 1. Additional baseline variables and extended laboratory results are provided in Table S2 in Multimedia Appendix 1.

**Table 1:** Baseline characteristics of patients with stroke stratified by admission timing.

Characteristics^a^		All participants (N=1327)	Onset ≤24 hours (n=696)	Onset >24 hours (n=631)	*P* value^b^	
Age (y), mean (SD)		63 (12.68)	66.03 (12.20)	59.66 (12.38)	<.001	
Age >65 y, n (%)		633 (47.70)	414 (59.48)	219 (34.71)	**<**.001	
**Sex, n (%)**						.28
	Male	933 (70.31)	480 (68.97)	453 (71.79)		
	Female	394 (29.69)	216 (31.03)	178 (28.21)		
**Region, n (%)**						<.001
	Urban	872 (79.42)	569 (82.94)	303 (73.54)		
	Rural	226 (20.58)	117 (17.06)	109 (26.46)		
**Medical history, n (%)**						
	History of hypertension	942 (70.99)	503 (72.27)	439 (69.57)	.30	
	History of diabetes	502 (37.83)	289 (41.52)	213 (33.76)	.005	
	History of dyslipidemia	146 (11.00)	93 (13.36)	53 (8.40)	.005	
	History of atrial fibrillation	122 (9.19)	89 (12.79)	33 (5.23)	<.001	
	History of carotid stenosis	49 (3.69)	44 (6.32)	5 (0.79)	<.001	
	History of cancer	53 (3.99)	43 (6.18)	10 (1.58)	<.001	
**Family history, n (%)**						
	Family history of ischemic stroke	70 (5.28)	16 (2.30)	54 (8.56)	<.001	
	Family history of hemorrhagic apoplexy	30 (2.26)	5 (0.72)	25 (3.96)	<.001	
	Family history of hypertension	104 (7.84)	14 (2.01)	90 (14.26)	<.001	
	Family history of diabetes	44 (3.32)	10 (1.44)	34 (5.39)	<.001	
**Admission comorbidities, n (%)**						
	Hypertension	701 (52.83)	494 (70.98)	207 (32.81)	<.001	
	Diabetes	385 (29.01)	295 (42.39)	90 (14.26)	<.001	
	Dyslipidemia	236 (17.78)	119 (17.10)	117 (18.54)	.52	
	Arteriosclerosis	160 (12.06)	57 (8.19)	103 (16.32)	<.001	
	Middle cerebral artery occlusion	106 (7.99)	87 (12.50)	19 (3.01)	<.001	
	Hyperhomocysteinemia	101 (7.61)	49 (7.04)	52 (8.24)	.48	
	Vertebral artery stenosis	187 (14.09)	157 (22.56)	30 (4.75)	<.001	
	Vertebral artery occlusion	64 (4.82)	55 (7.90)	9 (1.43)	<.001	
	Carotid artery stenosis	191 (14.39)	138 (19.83)	53 (8.40)	<.001	
	Hemiplegia	1246 (93.90)	672 (96.55)	574 (90.97)	<.001	
	Dysphagia	448 (33.76)	211 (30.32)	237 (37.56)	.007	
	Sensory loss	232 (17.48)	91 (13.07)	141 (22.35)	<.001	
	Motor disorder	685 (51.62)	438 (62.93)	247 (39.14)	<.001	
	Speech disorder	685 (51.62)	339 (48.71)	346 (54.83)	.03	
	Cognitive impairment	341 (25.70)	113 (16.24)	228 (36.13)	<.001	

^a^Data are mean (SD), median (IQR), or n (%).

^b^*P* values are from chi-square test, Fisher exact test, Student *t* test, or Mann–Whitney *U* test, as appropriate.

Significant demographic differences were observed between the 2 cohorts. Patients in the early-arrival group (≤24 h) were significantly older (mean age: 66.03, SD 12.20 vs 59.66, SD 12.38 years; *P*<.001) and were more likely to be married (677/696, 97.27% vs 580/630, 92.06%; *P*<.001) and urban residents (569/686, 82.94% vs 303/412, 73.54%; *P*<.001). Social factors also played a role, as early arrivals had a higher proportion of used individuals and more children. Lifestyle factors showed a significant association with admission timing (*P*<.001). A substantial majority of early arrivals were either current or former smokers (226/246, 91.87%) and drinkers (201/251, 80.08%), whereas over half of the late-arrival group reported never smoking (277/500, 55.40%) or never drinking (285/542, 52.58%).

Clinical presentations at onset significantly influenced the speed of hospital presentation. Ischemic stroke was almost universal in the early-arrival group (693/696, 99.57%) compared to the late group (468/631, 74.17%; *P*<.001). Patients with a personal medical history of atrial fibrillation, carotid stenosis, and cancer were significantly more likely to arrive within 24 hours. Patients with a family history of ischemic stroke, hypertension, or diabetes were more prevalent in the delayed-admission group (*P*<.001). Upon admission, the early-arrival group presented with higher rates of hypertension (494/696, 70.98%) and diabetes (295/696, 42.39%). In contrast, the late-arrival group exhibited significantly higher frequencies of cognitive impairment (228/631, 36.13% vs 113/696, 16.24%), sensory loss (141/631, 22.35% vs 91/696, 13.07%), and dysphagia (237/631, 37.56% vs 211/696, 30.32%), suggesting that functional and cognitive barriers may contribute to prehospital delays.

Physiological parameters at admission further differentiated the groups. The early-arrival group presented with significantly higher blood pressure (Systolic: 141.94 vs 136.16 mm Hg; Diastolic: 84.17 vs 82.04 mm Hg; *P*<.001) and elevated lactate dehydrogenase (LD) levels (224.02 vs 191.56 U/L; *P*<.001). Laboratory tests revealed that late arrivals had significantly higher levels of uric acid (UA: 309.22 vs 276.72 umol/L; *P*<.001) and lower albumin (ALB: 38.62 vs 40.35 g/L; *P*=.01). Furthermore, the respiratory rate was higher in the late-arrival group (18.64 vs 18.09 bpm; *P*<.001).

### Model Performance and Comparative Analysis

**Figure 1:**
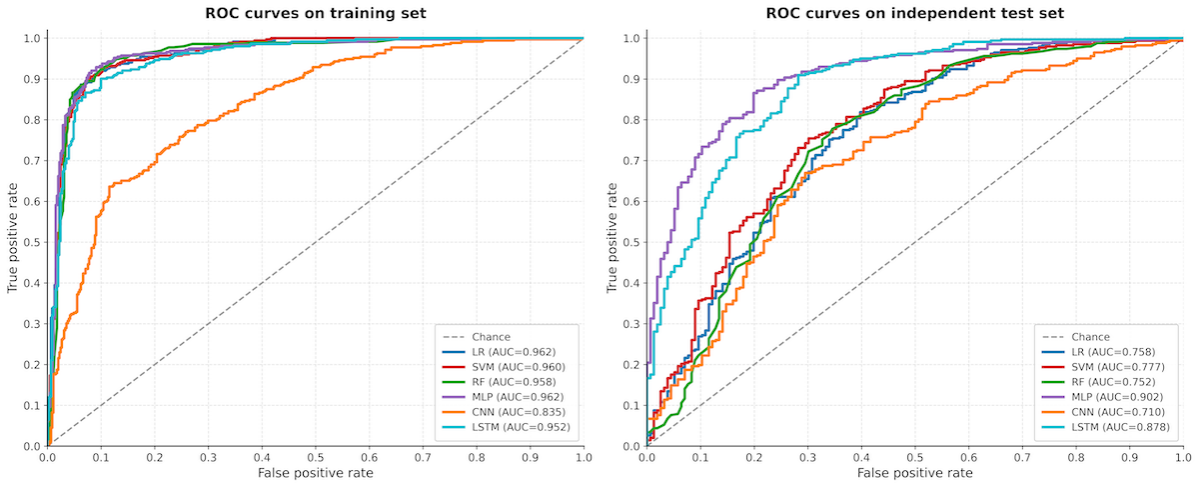
Receiver operating characteristic (ROC) curves of the six predictive models for early hospital admission. (A) Model performance within the train set (2019-2022); (B) Model performance within the independent temporal testing set (2023-2025). AUC: area under the receiver operating characteristic curve; CNN: convolutional neural network; LR: logistic regression; LSTM: long short-term memory; MLP: multilayer perceptron; RF: random forest; ROC: receiver operating characteristic; SVM: support vector machine.

Across the 6 candidate models, DL approaches generally showed better performance than the conventional ML models in the independent temporal testing set (Table 2; Figure 1). Among all models, the MLP achieved the highest overall performance, with an AUC of 0.9020 (95% CI 0.8718-0.9283), sensitivity of 0.9152, specificity of 0.7564, and *F*_1_-score of 0.9033 in the independent temporal testing set. The LSTM network also showed relatively strong discrimination (AUC 0.8778), whereas the 1D-CNN showed lower overall performance (AUC 0.7105). LR, SVM, and RF demonstrated lower performance in the independent temporal testing set, with AUC values of 0.7576, 0.7769, and 0.7523, respectively.

**Table 2:** Comparison of predictive performance across machine learning and deep learning models.

Model			AUC^a^		Accuracy		Precision		Sensitivity		Specificity		*F*_1_-score
**Train set (n=821)**													
	MLP^b^	0.9617		*0.9165^c^*		0.9154		*0.9170*		0.9170		*0.9154*	
	LSTM^d^	0.9519		0.8941		0.8928		0.8933		0.8933		0.8926	
	CNN^e^	0.8351		0.7572		0.7848		0.7537		0.7537		0.7462	
	RF^f^	0.9583		0.9140		*0.9346*		0.8663		*0.9511*		0.8980	
	SVM^g^	0.9601		0.9078		0.8860		0.9090		0.9069		0.8965	
	LR^h^	*0.9619*		0.9103		0.8906		0.9090		0.9113		0.8991	
**Temporal testing set (n=506)**													
	MLP	*0.9020*		*0.8654*		*0.8917*		*0.9152*		0.7564		*0.9033*	
	LSTM	0.8778		0.8434		0.8568		0.9069		0.6603		0.8904	
	CNN	0.7105		0.6506		0.8230		0.6257		0.7051		0.7109	
	RF	0.7523		0.7149		0.8401		0.7222		0.6987		0.7767	
	SVM	0.7769		0.6305		0.8726		0.5409		*0.8269*		0.6679	
	LR	0.7576		0.6124		0.8465		0.5322		0.7885		0.6535	

^a^AUC: area under the receiver operating characteristic curve.

^b^MLP: multilayer perceptron.

^c^Italicized values indicate the best performance among the compared models for each metric.

^d^LSTM: long short-term memory.

^e^CNN: convolutional neural network.

^f^RF: random forest.

^g^SVM: support vector machine.

^h^LR: logistic regression.

Formal statistical comparisons supported the superior discrimination of the MLP model. Compared with the alternative models, the MLP achieved significantly higher AUC values than LR (ΔAUC=0.1444; FDR-adjusted *P*<.001), SVM (ΔAUC=0.1251; FDR-adjusted *P*<.001), RF (ΔAUC=0.1497; FDR-adjusted *P*<.001), and 1D-CNN (ΔAUC=0.1916; FDR-adjusted *P*<.001). The improvement over the LSTM was smaller but remained statistically significant (ΔAUC=0.0243; FDR-adjusted *P*=.03). At the primary operating threshold of 0.5, McNemar tests showed that the MLP made significantly fewer classification errors than LR, SVM, RF, and 1D-CNN after multiple-comparison correction, whereas the difference between the MLP and LSTM was not statistically significant (FDR-adjusted *P*=.65).

From a clinical perspective, sensitivity is particularly important for identifying patients at risk of delayed hospital presentation. In this regard, the MLP maintained a sensitivity of 91.52% in the independent temporal testing set, markedly higher than that of LR and SVM. In a supplementary comparison, the LASSO-selected model achieved slightly higher discrimination and markedly better sensitivity than the full-feature model in the independent temporal testing set (AUC 0.9020 vs 0.8894; sensitivity 0.9152 vs 0.8452), while using fewer predictors, supporting the parsimony of the final modeling strategy (Table S3 in Multimedia Appendix 1). Detailed CIs and pairwise DeLong and McNemar comparisons are provided in Tables S4-S6 in Multimedia Appendix 1.

### Calibration and Clinical Utility Analysis

**Figure 2:**
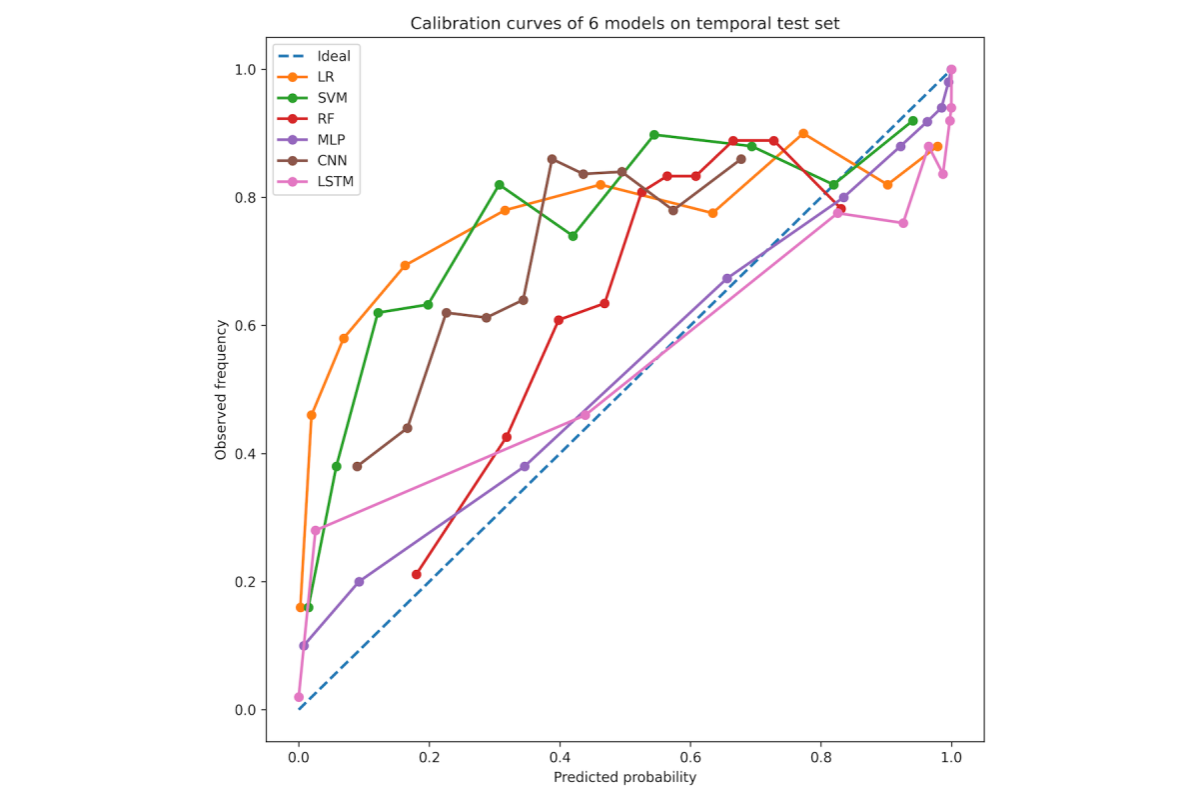
Calibration curves of the six predictive models in the independent temporal testing set CNN: convolutional neural network; LR: logistic regression; LSTM: long short-term memory; MLP: multilayer perceptron; RF: random forest; SVM: support vector machine.

We further evaluated model calibration and clinical utility in the independent temporal testing set. Among the 6 candidate models, the MLP showed the most favorable calibration profile, with the lowest Brier score (0.1153) and a calibration curve closest to the ideal reference line (Table 3; Figure 2). However, calibration showed some room for improvement: the calibration intercept was 0.279, and the calibration slope was 0.600, suggesting mild overall risk underestimation and some deviation from ideal probability calibration.

**Table 3:** Calibration summary.

Model	Brier score	Calibration intercept	Calibration slope
MLP^a^	0.11526	0.279164	0.600148
LSTM^b^	0.130984	0.386194	0.332867
CNN^c^	0.29329	1.44807	0.875987
RF^d^	0.202934	0.826486	1.21653
SVM^e^	0.269879	1.302286	0.521936
LR^f^	0.2832	1.222271	0.354217

^a^MLP: multilayer perceptron.

^b^LSTM: long short-term memory.

^c^CNN: convolutional neural network.

^d^RF: random forest.

^e^SVM: support vector machine.

^f^LR: logistic regression.

**Figure 3:**
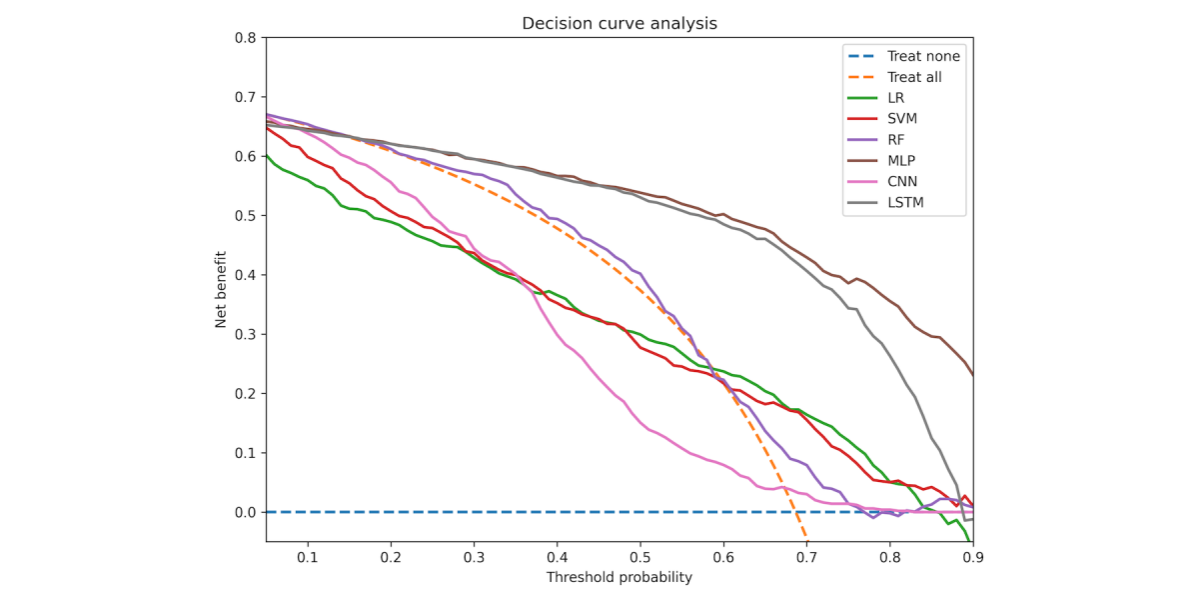
Decision curve analysis of the six predictive models in the independent temporal testing set. CNN: convolutional neural network; LR: logistic regression; LSTM: long short-term memory; MLP: multilayer perceptron; RF: random forest; SVM: support vector machine.

Decision curve analysis showed that the MLP generally provided greater net benefit than the alternative models across a range of clinically relevant threshold probabilities, supporting its potential clinical utility beyond conventional discrimination metrics (Figure 3). In the primary analysis, a default threshold of 0.5 was used for binary classification. Additional threshold sensitivity analyses showed that the MLP maintained a favorable balance between sensitivity and specificity across representative cutoffs of 0.3, 0.4, 0.5, and 0.6, with the threshold of 0.5 providing the most balanced overall operating point in the independent Temporal Testing Set (Figure S1 and Table S7 in Multimedia Appendix 1).

### Interpretability and Temporal Stability of Feature Contributions

**Figure 4:**
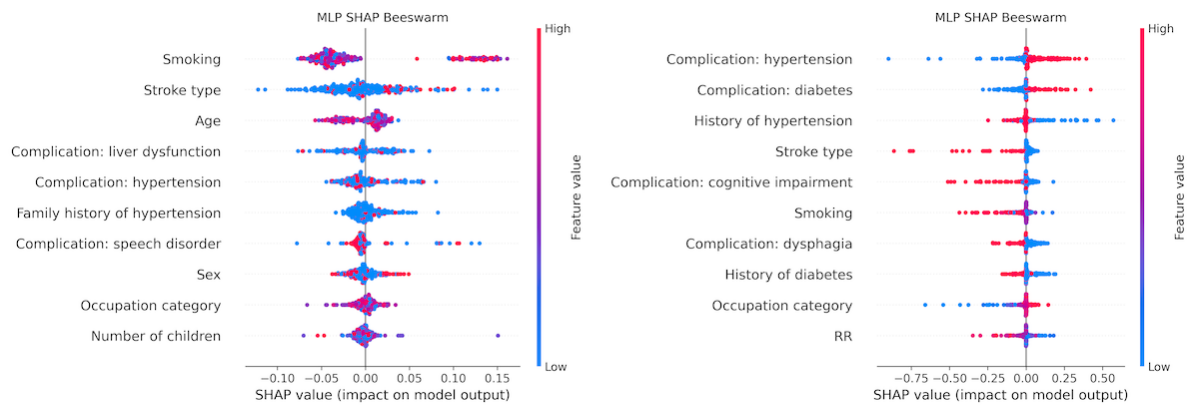
Shapley additive explanations beeswarm plots for the multilayer perceptron model in the train set and the independent temporal testing set. (A) Train set (2019-2022); (B) independent temporal testing set (2023-2025). Each point represents an individual patient, with color indicating the feature value (red for high, blue for low) and position on the x-axis representing the contribution of that feature to the predicted probability of early hospital admission (≤24 h). Feature values shown in the beeswarm plots represent preprocessed model inputs rather than raw variable coding; continuous variables are displayed on their standardized scale after z-score normalization, and binary variables such as dysphagia are shown according to the encoded values used in the final model input. MLP: multilayer perceptron; SHAP: Shapley additive explanations.

To improve the interpretability of the MLP model and assess the temporal stability of feature contributions, we performed SHAP analysis in both the train set and the independent temporal testing set (Figure 4). In the train set, the model was influenced by a combination of demographic, clinical, and complication-related features. Smoking, stroke type, age, liver dysfunction, and admission hypertension ranked among the most important contributors, whereas family history of hypertension, speech disorder, sex, occupation category, and number of children also contributed to the model output. These findings indicate that, during model development, prediction reflected not only acute clinical status but also broader demographic and contextual characteristics. Because SHAP analysis was based on the final preprocessed model inputs, the feature values shown in the SHAP plots should be interpreted on the model-input scale rather than as raw variable values recorded in the medical records.

In the independent temporal testing set, the SHAP pattern shifted toward more overt acute clinical features. Admission hypertension and diabetes emerged as the leading predictors, followed by history of hypertension, stroke type, cognitive impairment, smoking, dysphagia, history of diabetes, occupation category, and respiratory rate. Dysphagia remained among the top predictors, and the feature-specific SHAP dependence plot showed that higher dysphagia values, corresponding to the presence of dysphagia after preprocessing, were predominantly associated with negative SHAP values for the positive class, indicating a lower predicted probability of early hospital admission. This pattern was consistent with the baseline descriptive analysis, in which dysphagia was more frequent among patients with delayed admission. At the same time, local SHAP values showed that the contribution of dysphagia was not uniform across all individuals, indicating that its effect should be interpreted within the broader nonlinear multivariable structure of the model rather than as an isolated deterministic signal. Overall, although the relative ranking of predictors changed from the train set to the independent temporal testing set, the model retained clinically coherent feature contributions over time. The dysphagia dependence plot, representative local waterfall plots, and background-sensitivity analyses are presented in Figure S2, Figure S3, and Table S8 in Multimedia Appendix 1.

### Subgroup Performance, Feature Drift, and Error Analysis Under Temporal Validation

Additional analyses were conducted in the independent temporal testing set to further characterize temporal performance patterns of the MLP, including subgroup performance analysis, feature-drift assessment, and prediction-error analysis. Subgroup analysis showed that the MLP maintained generally strong discrimination across major demographic and clinical strata. The AUC was 0.903 in patients aged ≤65 years and 0.883 in those aged >65 years, and was 0.905 in male patients and 0.895 in female patients. Performance was also favorable in clinically relevant subgroups, including patients with dysphagia (AUC 0.944; sensitivity 0.955), admission hypertension (AUC 0.919; sensitivity 0.942), and admission diabetes (AUC 0.902; sensitivity 0.972), although these subgroup results should be interpreted cautiously because some strata were relatively small.

Feature-drift analysis showed that the temporal shift between the train set and the independent Temporal Testing Set was selective rather than global. After false discovery rate correction, the most notable changes involved several physiological and complication-related variables, including CKMB (standardized mean difference [SMD] = −0.463), admission hypertension (prevalence difference = +0.124), respiratory rate (SMD = +0.110), liver dysfunction (prevalence difference = −0.053), and family history of hypertension (prevalence difference = −0.034). These findings indicate that temporal change was concentrated in specific clinical domains rather than reflecting a complete restructuring of the predictor space.

Prediction-error analysis further showed that residual misclassification was clinically patterned. Among patients with true early admission, false-negative cases were less likely than true-positive cases to present with admission hypertension (45.5% vs 79%) or diabetes (12.1% vs 45%), and they had higher uric acid levels (median 312, IQR 271-389 vs 255, IQR 198-313). Among delayed-admission cases, false-positive predictions differed most clearly from true-negative cases in stroke type, whereas other differences were smaller and less consistent after multiple-comparison correction. Detailed subgroup, drift, and error-analysis results are provided in Tables S9-S11 in Multimedia Appendix 1.

## Discussion

### Principal Findings

This multicenter retrospective study developed and temporally validated a prediction framework for early hospital admission after acute stroke using routinely collected clinical data from 6 hospitals in China. Within this multicenter Chinese cohort, the MLP showed the most favorable overall performance in the independent temporal testing set. Additional analyses of calibration, SHAP-based interpretation, and temporal degradation patterns provided further insight into the strengths and remaining limitations of the model.

Our findings delineate distinct phenotypic profiles between early presenters and patients with delayed admission. Individuals arriving within the therapeutic window were generally older and more likely to reside in urban areas. This observation is consistent with national registry evidence indicating that older adults often benefit from established health care networks and heightened vigilance related to multimorbidity, whereas younger populations and rural residents encounter structural barriers and lower stroke literacy, resulting in prolonged onset-to-door times [33,34]. The pronounced urban-rural disparity underscores persistent inequities in EMS accessibility and the uneven coverage of “Stroke Map” networks, with transport logistics remaining a critical bottleneck in rural regions [35,36]. Clinically, the higher prevalence of cognitive impairment and sensory deficits among delayed presenters supports the “symptom imperceptibility” hypothesis, whereby anosognosia or nonmotor manifestations attenuate symptom recognition and postpone EMS activation [37].

This study showed the most favorable overall performance of the MLP in predicting early hospitalization risk. Its optimal performance on the independent temporal testing set was consistent with our study hypothesis that DL excels at capturing complex nonlinear interactions among clinical variables. In sharp contrast to traditional linear models, which exhibited a marked decline in temporal validation performance and a substantial decline in sensitivity during temporal extrapolation, the MLP demonstrated robust generalization capabilities [38,39]. Furthermore, due to its global feature processing mechanism, the MLP outperformed the CNN in handling nonspatial tabular data. Critically, the MLP maintained a high sensitivity of 91% in the testing cohort, establishing its suitability as a prehospital screening tool where the prioritization of low false-negative rates is essential to ensure high-risk patients are not overlooked. Combined with the interpretability support provided by SHAP analysis, these findings indicate that the proposed architecture possesses potential clinical utility. Additional temporal analyses suggested that the relative robustness of the MLP was associated with selective rather than global temporal drift. The model maintained strong performance in clinically relevant subgroups, particularly among patients with dysphagia, admission hypertension, and admission diabetes. This pattern is broadly consistent with previous evidence showing that more severe or more readily recognizable stroke presentations are associated with earlier hospital arrival, whereas atypical presentations may be more vulnerable to delayed recognition [40,41]. Residual errors were also clinically patterned rather than random: false-negative early-admission cases were less likely to exhibit overt physiological warning signs, whereas some false-positive delayed-admission cases shared characteristics with early presenters.

SHAP analysis further contextualized these findings by revealing temporally evolving yet clinically coherent feature contributions. In the train set, model predictions were influenced not only by clinical impairments but also by system-level and social-contextual factors. Hospital-level and family-related variables, for example, underscored the potential roles of health care accessibility and social support in shaping early help-seeking behavior [42]. Dysphagia also remained an important predictor across analyses. In the independent temporal testing set, higher dysphagia values were generally associated with a lower predicted probability of early hospital admission. However, this effect was not strictly uniform at the individual level, indicating that the contribution of dysphagia should be interpreted within the broader nonlinear multivariable structure of the model rather than as an isolated deterministic signal. This finding may reflect delayed recognition of atypical or posterior-circulation-related stroke presentations in some patients, because posterior circulation strokes may present with less typical prehospital symptom patterns and may therefore be more vulnerable to delayed identification and hospital presentation [43,44]. However, this interpretation should be considered cautiously and warrants further confirmation with richer clinical and imaging data. Meanwhile, the model’s reliance on the independent temporal testing set shifted toward more overt physiological abnormalities, with admission hypertension and diabetes emerging as dominant predictors of early presentation. This pattern suggests that acute, measurable physiological derangements may function as immediate triggers for EMS activation [45]. The persistence of dysphagia and stroke type across both periods further supports the clinical stability of these predictors and reinforces the temporal robustness of the model.

The integration of an MLP-based framework into prehospital stroke systems may support more targeted risk stratification for patients at risk of delayed presentation. Because all predictors were derived from routinely available electronic medical record data, the model may be scalable in digitally enabled clinical settings. Beyond binary classification, the framework also provides a more granular risk profile of patients vulnerable to prehospital delay. Through SHAP-based interpretation, predictors such as dysphagia and other clinical or contextual factors may help identify patient groups who are less likely to present promptly after stroke onset. These insights could inform more targeted public health strategies, rather than relying solely on broad, non-specific stroke awareness campaigns, and may be relevant for improving resource allocation in high-risk populations. At the same time, although the MLP demonstrated the best overall calibration profile among the candidate models, calibration was not fully optimal, and recalibration may be necessary before implementation in deployment-oriented settings. In addition, although the MLP showed favorable performance under temporal validation, this study does not constitute true geographic external validation because all participating hospitals were located within the same national health care system. Further validation across independent institutions, regions, and health care settings will therefore be necessary before broader implementation can be considered.

Several limitations should be acknowledged. First, the retrospective design of this study precludes the complete elimination of information bias. In addition, we did not formally evaluate the underlying missing-data mechanism (eg, missing completely at random, missing at random, or missing not at random), and some residual bias related to missingness cannot be excluded. The accuracy of symptom-onset time also depended on retrospectively documented medical records and was typically based on patient or caregiver recall at admission, which may have introduced imprecision in the onset-to-admission interval, particularly in wake-up strokes or in individuals with cognitive impairment. Second, the current model was developed exclusively using structured electronic medical record data. Although this enhances feasibility for routine clinical implementation, the absence of imaging-derived variables limits the model’s ability to directly capture lesion location and radiological severity, including clinically important patterns such as posterior circulation involvement. This limitation may partly explain why some clinical proxies, such as dysphagia, emerged as influential predictors. Third, despite the use of SHAP to improve interpretability, neural networks still retain residual “black-box” characteristics, and the high-dimensional feature interactions learned by the MLP may not be fully transparent to clinicians. Although the use of an independent temporal testing cohort helped mitigate concerns regarding optimistic bias, we did not implement a formal nested cross-validation framework. In addition, while the present sample size was sufficient to support model development and independent temporal validation, larger prospective multicenter datasets are still needed for further refinement and validation of DL models. Future research should therefore prioritize prospective external validation, multimodal data integration, including imaging and prehospital process metrics, and the development of clinician-centered explanation interfaces to improve interpretability and clinical acceptability.

### Conclusions

In this multicenter retrospective study, the MLP showed favorable temporal performance for predicting early hospital admission (≤24 h) after acute stroke within a multicenter Chinese cohort. SHAP-based interpretation suggested that dysphagia, admission hypertension, diabetes, and stroke type were important contributors to model predictions. These findings may support future risk stratification and more targeted public health interventions, although external validation, recalibration, and further prospective evaluation remain necessary before deployment-oriented application.

## Data Availability

The datasets analyzed during this study are not publicly available due to patient privacy and hospital regulations, but are available from the corresponding author on reasonable request. The code used for data preprocessing, model development, validation, and Shapley additive explanations (SHAP) analysis is publicly available in the GitHub repository [46].

## Appendix

Multimedia Appendix 1 Preprocessing sensitivity analyses, feature selection, cross-validation, calibration, statistical comparison, SHAP (Shapley additive explanations) interpretation, temporal degradation analyses, and code-related documentation.

## Abbreviations

AUC: area under the receiver operating characteristic curve
CNN: convolutional neural network
DL: deep learning
EMS: emergency medical service
ICD-11: International Classification of Diseases, 11th Revision
ICF: International Classification of Functioning, Disability and Health
LASSO: least absolute shrinkage and selection operator
LR: logistic regression
LSTM: long short-term memory
ML: machine learning
MLP: multilayer perceptron
1D-CNN: one-dimensional convolutional neural network
RF: random forest
SHAP: Shapley additive explanations
SVM: support vector machine
