# Supplementary material for: Prediction of Early Hospital Admission (≤24 Hours) After Stroke Using Machine Learning and Deep Learning: Multicenter Study From China
Source: JMIR Med Inform. 2026 Jun 24;14:e90852. doi: 10.2196/90852 (PMC13347082; doi:10.2196/90852)
Supplement: Multimedia Appendix 1 [file medinform_v14i1e90852_app1.docx]

**Multimedia Appendix 1. Supplementary Tables and Figures**

Table S1. Sensitivity analysis of alternative imputation strategies for the MLP in the independent Temporal Testing Set

| **Model** | **Accuracy** | **Precision** | **Sensitivity** | **Specificity** | **AUC** | **F1-Score** |
| --- | --- | --- | --- | --- | --- | --- |
| KNN(K=5) | **0.8654** | 0.8917 | **0.9152** | 0.7564 | 0.9020 | **0.9033** |
| KNN(K=3) | 0.8431 | 0.8683 | 0.9064 | 0.6987 | 0.8938 | 0.8869 |
| KNN(K=7) | 0.8514 | 0.8828 | 0.9035 | 0.7371 | **0.9036** | 0.8930 |
| MICE | 0.8437 | **0.9069** | 0.8666 | **0.7894** | 0.8847 | 0.8863 |
| MEAN | 0.8333 | 0.8768 | 0.8897 | 0.6964 | 0.8698 | 0.8832 |

Table S2. Extended baseline characteristics of stroke patients stratified by admission timing

| **Characteristics** | **All participants (N=1327)** | **Onset ≤ 24 hours (n=696)** | **Onset > 24 hours (n=631)** | **p value** |
| --- | --- | --- | --- | --- |
| Married, n (%) | 1257 (94.80) | 677 (97.27) | 580 (92.06) | **<0.001** |
| Number of children (IQR) | 2.00 (1.00–3.00) | 2.00 (2.00–3.00) | 2.00 (1.00–2.00) | **<0.001** |
| Ischemic stroke, n (%) | 1161 (87.49) | 693 (99.57) | 468 (74.17) | **<0.001** |
| Occupation, n (%) |  |  |  | **<0.001** |
| In employment | 960 (72.51) | 544 (78.27) | 416 (66.14) |  |
| Retired | 306 (23.11) | 144 (20.72) | 162 (25.76) |  |
| Unemployed | 58 (4.38) | 7 (1.01) | 51 (8.11) |  |
| Smoking, n (%) |  |  |  | **<0.001** |
| Former Smoker | 149 (19.97) | 79 (32.11) | 70 (14.00) |  |
| Current Smoker | 300 (40.21) | 147 (59.76) | 153 (30.60) |  |
| Never Smoked | 297 (39.81) | 20 (8.13) | 277 (55.40) |  |
| Drinking, n (%) |  |  |  | **<0.001** |
| Former Drinker | 106 (13.37) | 53 (21.12) | 53 (9.78) |  |
| Current Drinker | 352 (44.39) | 148 (58.96) | 204 (37.64) |  |
| Never Drank | 335 (42.24) | 50 (19.92) | 285 (52.58) |  |
| Admission comorbidities, n (%) | | | | |
| depression | 69 (5.20) | 46 (6.61) | 23 (3.65) | **0.021** |
| anxiety | 74 (5.58) | 49 (7.04) | 25 (3.96) | **0.017** |
| vestibular balance disorder | 19 (1.43) | 0 (0.00) | 19 (3.01) | **<0.001** |
| ataxia | 41 (3.09) | 26 (3.74) | 15 (2.38) | 0.215 |
| hyperuricemia | 67 (5.05) | 44 (6.32) | 23 (3.65) | **0.033** |
| liver dysfunction | 133 (10.02) | 43 (6.18) | 90 (14.26) | **<0.001** |
| shoulder subluxation | 48 (3.62) | 4 (0.57) | 44 (6.97) | **<0.001** |
| pulmonary infection | 315 (23.74) | 222 (31.90) | 93 (14.74) | **<0.001** |
| upper GI bleeding | 42 (3.17) | 39 (5.60) | 3 (0.48) | **<0.001** |
| Laboratory test, mean (SD) | | | | |
| GLU (mmol/L) | 5.93 (2.04) | 5.86 (1.94) | 6.02 (2.16) | 0.207 |
| Na (mmol/L) | 140.06 (5.35) | 140.29 (3.61) | 139.76 (7.00) | 0.125 |
| K (mmol/L) | 4.54 (14.90) | 5.03 (19.78) | 3.91 (0.42) | 0.151 |
| Cl (mmol/L) | 102.70 (8.54) | 102.78 (9.71) | 102.60 (6.69) | 0.700 |
| Ca (mmol/L) | 2.31 (1.10) | 2.25 (0.14) | 2.39 (1.63) | 0.075 |
| ALB (g/L) | 39.36 (12.84) | 38.62 (16.44) | 40.35 (5.02) | **0.012** |
| A/G | 1.86 (7.25) | 1.50 (0.29) | 2.32 (11.00) | 0.103 |
| UA (umol/L) | 290.11 (96.86) | 276.72 (94.61) | 309.22 (96.93) | **<0.001** |
| LPa (mmol/L) | 339.58 (348.27) | 355.08 (354.52) | 278.11 (315.92) | **0.010** |
| CHO (mmol/L) | 3.89 (6.24) | 3.93 (8.20) | 3.83 (1.42) | 0.761 |
| HDL (mmol/L) | 1.03 (0.40) | 1.07 (0.49) | 0.98 (0.24) | **<0.001** |
| LDL (mmol/L) | 2.48 (8.86) | 2.51 (11.52) | 2.44 (2.68) | 0.886 |
| TG (mmol/L) | 1.39 (0.67) | 1.34 (0.68) | 1.46 (0.66) | **0.003** |
| P (mmol/L) | 1.21 (0.24) | 1.18 (0.25) | 1.24 (0.22) | **<0.001** |
| Mg (mmol/L) | 0.95 (1.60) | 1.03 (2.16) | 0.86 (0.09) | 0.064 |
| Fe (umol/L) | 13.62 (11.72) | 13.63 (12.71) | 13.58 (5.06) | 0.946 |
| CO2CP (mmol/L) | 23.77 (3.50) | 19.97 (6.48) | 24.05 (3.01) | **0.001** |
| LD (U/L) | 211.16 (72.22) | 224.02 (70.31) | 191.56 (70.76) | **<0.001** |
| CRP (mg/L) | 8.70 (17.02) | 8.97 (17.80) | 7.78 (14.06) | 0.431 |
| AMY (U/L) | 59.51 (35.24) | 59.30 (37.52) | 60.37 (24.22) | 0.725 |
| SBP (mmHg) | 139.22 (19.58) | 141.94 (20.25) | 136.16 (18.35) | **<0.001** |
| DBP (mmHg) | 83.17 (11.70) | 84.17 (11.95) | 82.04 (11.32) | **<0.001** |
| RR (bpm) | 18.35 (2.25) | 18.09 (1.57) | 18.64 (2.79) | **<0.001** |

*Note: Data are mean (SD), median (IQR), or n (%). p values are from χ² test, Fisher’s exact test, Student’s t test, or Mann–Whitney U test, as appropriate.*

Table S3. Performance comparison between the full-feature model and the LASSO-selected model in the independent Temporal Testing Set

| **Model** | **Accuracy** | **Precision** | **Sensitivity** | **Specificity** | **AUC** | **F1-Score** |
| --- | --- | --- | --- | --- | --- | --- |
| Full feature | 0.8750 | 0.8525 | 0.8452 | 0.8452 | 0.8894 | 0.8487 |
| Selected feature | 0.8654 | **0.8917** | **0.9152** | 0.7564 | **0.9020** | **0.9033** |

Table S4. Confidence intervals for key performance metrics in the independent Temporal Testing Set

| **Model** | **AUC (95% CI)** | **Sensitivity (95% CI)** | **Specificity (95% CI)** | **F1-score (95% CI)** |
| --- | --- | --- | --- | --- |
| MLP | 0.9020 (0.8718–0.9283) | 0.9152 (0.8716–0.9327) | 0.7564 (0.6667–0.8037) | 0.9033 (0.8671–0.9158) |
| LSTM | 0.8778 (0.8419–0.9097) | 0.9069 (0.8968–0.9527) | 0.6603 (0.5786–0.7333) | 0.8904 (0.8648–0.9120) |
| CNN | 0.7105 (0.6573–0.7587) | 0.6257 (0.5836–0.6678) | 0.7051 (0.6654–0.7448) | 0.7109 (0.6714–0.7504) |
| RF | 0.7523 (0.6989–0.7993) | 0.7222 (0.6889–0.7830) | 0.6987 (0.5952–0.7434) | 0.7767 (0.7433–0.8149) |
| SVM | 0.7769 (0.7286–0.8221) | 0.5409 (0.4975–0.5843) | 0.8269 (0.7847–0.9007) | 0.6679 (0.5743–0.6714) |
| LR | 0.7576 (0.7077–0.8048) | 0.5322 (0.4789–0.5853) | 0.7885 (0.7189–0.8493) | 0.6535 (0.6056–0.6985) |

*Note: CI, confidence interval; MLP, multilayer perceptron; LSTM, long short-term memory; SVM, support vector machine; LR, logistic regression; RF, random forest; CNN, one-dimensional convolutional neural network.*

Table S5. Pairwise DeLong comparisons of AUC between the MLP model and alternative models in the independent Temporal Testing Set

| **Comparison** | **ΔAUC** | **DeLong *P* value** | **FDR-adjusted *P* value** |
| --- | --- | --- | --- |
| MLP vs LR | 0.1444 | 1.60 × 10^-8^ | 2.67 × 10^-8^ |
| MLP vs SVM | 0.1251 | 6.19 × 10^-7^ | 7.73 × 10^-7^ |
| MLP vs RF | 0.1497 | 1.26 × 10^-8^ | 2.67 × 10^-8^ |
| MLP vs CNN | 0.1916 | 1.12 × 10^-11^ | 5.62 × 10^-11^ |
| MLP vs LSTM | 0.0243 | 0.0274 | 0.0274 |

*Note: ΔAUC indicates the difference in AUC between the MLP model and the comparator model. P values were adjusted for multiple comparisons using the false discovery rate (FDR) procedure.*

Table S6. Pairwise McNemar comparisons between the MLP model and alternative models in the independent Temporal Testing Set

| **Comparison** | **McNemar statistic** | **McNemar *P* value** | **FDR-adjusted *P* value** |
| --- | --- | --- | --- |
| MLP vs LR | 74.4599 | 6.19 × 10^-18^ | 1.06 × 10^-17^ |
| MLP vs SVM | 74.4048 | 6.36 × 10^-18^ | 1.06 × 10^-17^ |
| MLP vs RF | 31.7956 | 1.71 × 10^-8^ | 2.14 × 10^-8^ |
| MLP vs CNN | 133.0830 | 8.67 × 10^-31^ | 4.34 × 10^-30^ |
| MLP vs LSTM | 0.2045 | 0.6511 | 0.6511 |

*Note: McNemar tests were conducted at the primary operating threshold of 0.5. P values were adjusted for multiple comparisons using the false discovery rate (FDR) procedure.*

Table S7. Comparison at representative thresholds

| **Threshold** | **Accuracy** | **Sensitivity** | **Specificity** | **PPV** | **NPV** | **F1** |
| --- | --- | --- | --- | --- | --- | --- |
| 0.3 | 0.8414 | 0.9386 | 0.6282 | 0.8470 | 0.8235 | 0.8904 |
| 0.4 | 0.8474 | 0.9181 | 0.6923 | 0.8674 | 0.7941 | 0.8920 |
| 0.5 | 0.8654 | 0.9152 | 0.7564 | 0.8829 | 0.7770 | 0.9033 |
| 0.6 | 0.8474 | 0.8713 | 0.7949 | 0.9030 | 0.7381 | 0.8869 |

Table S8. Background-sensitivity analysis of SHAP importance rankings for the MLP model

| **Repeated analysis** | **Top 10 features*** | **Mean absolute SHAP value for dysphagia** | **Median SHAP value for dysphagia** |
| --- | --- | --- | --- |
| 1 | Complication: hypertension; Complication: diabetes; IgG; Smoking; History of diabetes; History of hypertension; Complication: vertebral artery stenosis; BMI; CKMB; Complication: dysphagia | 0.035969 | 0.01227 |
| 2 | Complication: hypertension; Complication: diabetes; IgG; Smoking; History of diabetes; Stroke type; Complication: vertebral artery stenosis; BMI; History of hypertension; CKMB | 0.037159 | 0.01538 |
| 3 | Complication: hypertension; Complication: diabetes; IgG; Smoking; Stroke type; History of hypertension; History of diabetes; CKMB; BMI; Complication: dysphagia | 0.040468 | 0.017009 |

*Top 10 features are listed in descending order of mean absolute SHAP value for each repeated analysis.

Table S9. Subgroup performance of the MLP model in the independent Temporal Testing Set

| **Subgroup** | **Positive rate** | **AUC** | **Accuracy** | **Precision** | **Sensitivity** | **Specificity** | **F1-score** |
| --- | --- | --- | --- | --- | --- | --- | --- |
| Age ≤65 years | 0.569 | 0.903 | 0.833 | 0.828 | 0.893 | 0.755 | 0.859 |
| Age >65 years | 0.802 | 0.883 | 0.869 | 0.925 | 0.911 | 0.700 | 0.918 |
| Male | 0.672 | 0.905 | 0.851 | 0.886 | 0.893 | 0.763 | 0.889 |
| Female | 0.720 | 0.895 | 0.853 | 0.877 | 0.926 | 0.667 | 0.901 |
| Ischemic stroke present | 0.687 | 0.902 | 0.851 | 0.883 | 0.904 | 0.737 | 0.893 |
| Dysphagia absent | 0.667 | 0.878 | 0.819 | 0.859 | 0.871 | 0.714 | 0.865 |
| Dysphagia present | 0.721 | 0.944 | 0.907 | 0.920 | 0.955 | 0.784 | 0.937 |
| Cognitive impairment absent | 0.723 | 0.899 | 0.858 | 0.893 | 0.912 | 0.714 | 0.903 |
| Cognitive impairment present | 0.571 | 0.900 | 0.832 | 0.843 | 0.868 | 0.784 | 0.855 |
| Admission hypertension absent | 0.597 | 0.869 | 0.791 | 0.855 | 0.783 | 0.804 | 0.818 |
| Admission hypertension present | 0.721 | 0.919 | 0.875 | 0.891 | 0.942 | 0.700 | 0.916 |
| Admission diabetes absent | 0.640 | 0.897 | 0.823 | 0.867 | 0.854 | 0.768 | 0.861 |
| Admission diabetes present | 0.765 | 0.902 | 0.898 | 0.903 | 0.972 | 0.659 | 0.936 |

*Note: MLP, multilayer perceptron; AUC, area under the receiver operating characteristic curve. Positive rate refers to the proportion of early-admission cases within each subgroup.*

Table S10. Selected train-to-test feature drift between the Train Set and the independent Temporal Testing Set

| **Feature** | **Type** | **Train Set value** | **independent Temporal Testing Set value** | **Drift effect** | **FDR adjusted P value** |
| --- | --- | --- | --- | --- | --- |
| CKMB | Continuous | 14.05 (SD 36.29) | 2.14 (SD 2.95) | -0.463 | 0.011 |
| IgG | Continuous | 9.01 (SD 2.62) | 10.24 (SD 4.33) | 0.342 | 1.000 |
| PA | Continuous | 236.64 (SD 70.15) | 224.68 (SD 68.21) | -0.173 | 0.291 |
| Hospital level | Continuous | 1.03 (SD 0.17) | 1.01 (SD 0.09) | -0.167 | 1.000 |
| Smoking | Continuous | 1.99 (SD 0.70) | 2.08 (SD 0.68) | 0.134 | 1.000 |
| Occupation category | Continuous | 6.35 (SD 1.61) | 6.14 (SD 1.55) | -0.131 | 0.211 |
| LD | Continuous | 218.47 (SD 71.46) | 227.92 (SD 75.78) | 0.128 | 0.291 |
| Admission hypertension | Binary | 59.7% | 72.1% | 0.124 | 0.0002 |
| Respiratory rate | Continuous | 18.23 (SD 2.42) | 18.46 (SD 1.58) | 0.110 | 0.049 |
| BMI | Continuous | 25.27 (SD 8.77) | 24.57 (SD 3.65) | -0.104 | 1.000 |

*Note: For continuous variables, drift effect is expressed as the standardized mean difference; for binary variables, drift effect is expressed as the prevalence difference between the independent Temporal Testing Set and the Train Set. Positive values indicate higher values or higher prevalence in the independent Temporal Testing Set, whereas negative values indicate lower values or lower prevalence. Continuous variables are presented as mean (SD), and binary variables are presented as prevalence.*

Table S11. Prediction-error analysis of the MLP model in the independent Temporal Testing Set

Panel A. False-negative versus true-positive comparisons among true early-admission cases

| **Feature** | **Type** | **False-negative cases** | **True-positive cases** | **Effect size** | **FDR-adjusted P value** |
| --- | --- | --- | --- | --- | --- |
| Uric acid | Continuous | Median 312.0 | Median 255.0 | -0.750 | 0.002 |
| Admission hypertension | Binary | 45.5% | 79.0% | 0.335 | 0.001 |
| Admission diabetes | Binary | 12.1% | 45.0% | 0.329 | 0.002 |
| Hospital level | Continuous | Median 1.0 | Median 1.0 | -0.354 | 0.001 |
| Dysphagia | Binary | 18.2% | 40.8% | 0.226 | 0.098 |

Panel B. False-positive versus true-negative comparisons among delayed-admission cases

| **Feature** | **Type** | **False-positive cases** | **True-negative cases** | **Effect size** | **FDR-adjusted P value** |
| --- | --- | --- | --- | --- | --- |
| Stroke type | Encoded categorical | Distribution differed between groups | Distribution differed between groups | 0.909 | 0.003 |
| LD | Continuous | Median 222.0 | Median 185.0 | -0.704 | 0.214 |
| Occupation category | Encoded categorical | Median 7.0 | Median 6.0 | -0.655 | 0.131 |
| Smoking | Encoded categorical | Median 2.0 | Median 3.0 | 0.464 | 0.486 |
| Uric acid | Continuous | Median 269.5 | Median 300.5 | 0.454 | 0.486 |

*Note: Panel A compares false-negative and true-positive predictions among patients with true early admission. Panel B compares false-positive and true-negative predictions among patients with delayed admission. For continuous variables, effect size is expressed as the standardized mean difference; for binary variables, effect size is expressed as the prevalence difference. Encoded categorical variables were analyzed on the model-input coding scale used in the error-analysis outputs. Positive and negative values indicate the direction of difference between the two comparison groups.*


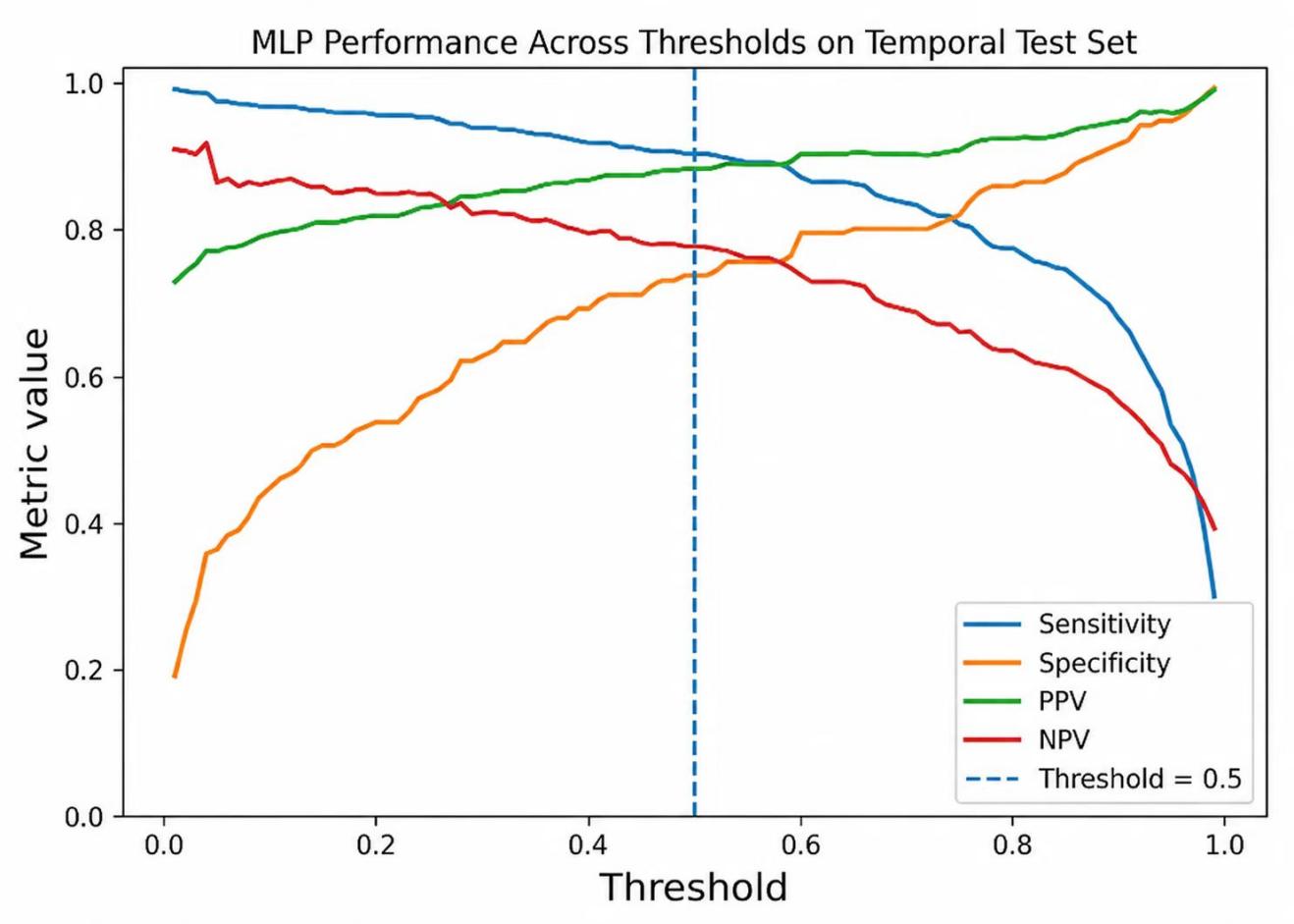


Figure S1. Model performance across a range of probability cutoffs


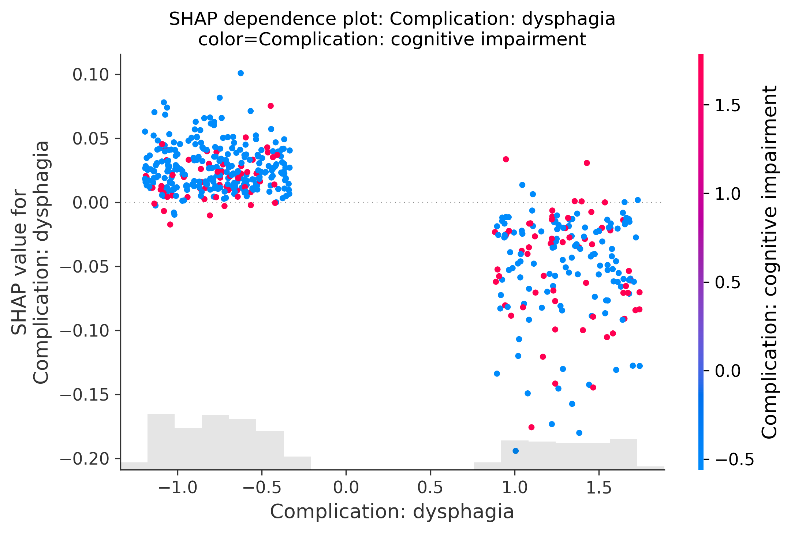


Figure S2. SHAP dependence plot for dysphagia in the external temporal testing cohort


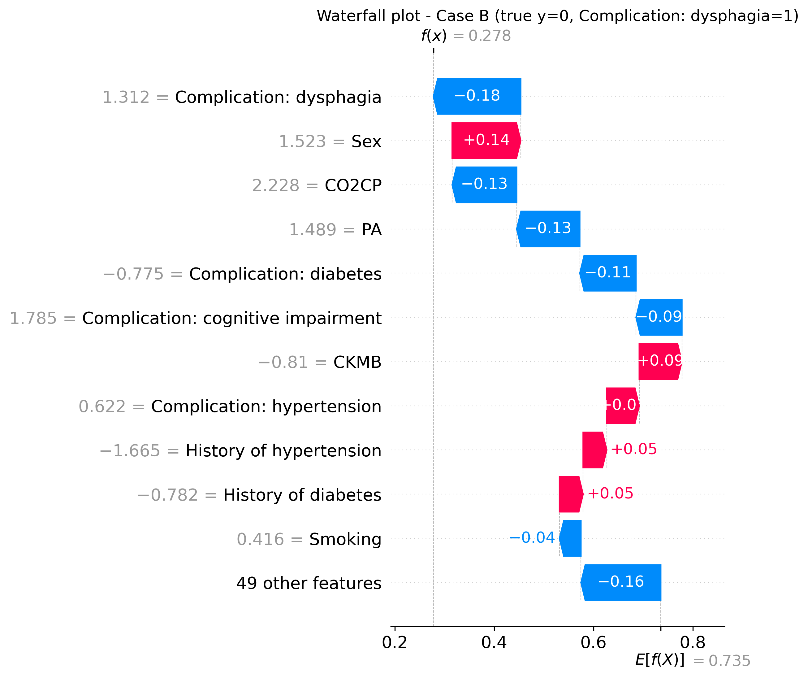


Figure S3. Representative SHAP waterfall plot for an individual delayed-admission case with dysphagia present
